# Supplementary material for: Smooth operator: Modifying the Anhøj rules to improve runs analysis in statistical process control
Source: PLoS One. 2020 Jun 4;15(6):e0233920. doi: 10.1371/journal.pone.0233920 (PMC7272012; doi:10.1371/journal.pone.0233920)
Supplement: Attachment — Submitted filename: Review of the paper PLOS.docx [file pone.0233920.s002.docx]

Review of the paper PONE-D-20-02344 titled

Smooth operator: Modifying the Anhøj rules to improve runs analysis in statistical process control

Summary:

In this paper, authors made an attempt to analyze the performance of a run chart using a run analysis. They consider joint distribution of crossing and run length and the likelihood method using R software - crossrun package. Diagnostic values of Anhoj rules were arrived.

Major Concerns:

Even though the problem the authors dealt in this paper is very interesting and have practical importance, the methodology of the proposed method is not explained clearly. For example, in method section, authors proposed likelihood ratio, without giving any details. Since this a statistical journal, author must clearly define the random variables C and L and it distribution and how the likelihood ratio arrived. Without this, the methodology proposed is not at all convincing.

Without a well explained methodology, it is hard to rate this paper.

Authors are encouraged to write down a clear methodology section and encouraged to re-submit the paper.
