# Supplementary material for: Smooth operator: Modifying the Anhøj rules to improve runs analysis in statistical process control
Source: PLoS One. 2020 Jun 4;15(6):e0233920. doi: 10.1371/journal.pone.0233920 (PMC7272012; doi:10.1371/journal.pone.0233920)
Supplement: Attachment — Submitted filename: crossrunbox_rebuttal.docx [file pone.0233920.s003.docx]

Re.: PONE-D-20-02344, Smooth operator: Modifying the Anhøj rules to improve runs analysis in statistical process control

Dear Editor,

Thank you for the opportunity to improve our manuscript. We have revised the article and we believe that we have fully addressed all points raised during the review process.

Here are our responses to the specific issues raised by the reviewers:

***Reviewer #1****: Even though the problem the authors dealt in this paper is very interesting and have practical importance, the methodology of the proposed method is not explained clearly. For example, in method section, authors proposed likelihood ratio, without giving any details. Since this a statistical journal, author must clearly define the random variables C and L and it distribution and how the likelihood ratio arrived. Without this, the methodology proposed is not at all convincing.

Without a well explained methodology, it is hard to rate this paper.*

**Response**: We have expanded the methods section detailing how to calculate specificity, sensitivity, and likelihood ratios of run chart rules. Also we have made it clear that theses measures have been explained in more detail in previous papers.

Also, reviewer #1 states that all data underlying the findings has NOT been made fully available. This we do not understand. All data including the R code to reproduce our findings are available in the supplementary material S1_crossrunbox.R.

***Reviewer #2****: this study demonstrates that it is possible to obtain better diagnostic properties of run charts by making minor adjustment to the critical values for C and L. Can this method work well if the distribution of C and L is not known?*

**Response**: The joint distribution of C and L is, in fact, known in our setting as described in our previous article (Wentzel-Larsen and Anhøj 2019, PLOS ONE). We have added a sentence clarifying this in the Discussion and conclusion section.

Kind regards

Jacob Anhøj & Tore Wentzel-Larsen
